# Secondhand smoke inhibits both Cl^- ^and K^+ ^conductances in normal human bronchial epithelial cells

**DOI:** 10.1186/1465-9921-10-120

**Published:** 2009-11-27

**Authors:** Amy N Savitski, Clementina Mesaros, Ian A Blair, Noam A Cohen, James L Kreindler

**Affiliations:** 1 Division of Pulmonary Medicine, The Children's Hospital of Philadelphia, Philadelphia, PA, USA; 2 Department of Pediatrics, University of Pennsylvania School of Medicine, Philadelphia, PA, USA; 3 Centers for Cancer Pharmacology and Excellence in Environmental Toxicology, Department of Pharmacology, University of Pennsylvania School of Medicine, Philadelphia, PA, USA; 4 Division of Rhinology, Department of Otorhinolaryngology, University of Pennsylvania School of Medicine, Philadelphia, PA, USA

## Abstract

Secondhand smoke (SHS) exposure is an independent risk factor for asthma, rhinosinusitis, and more severe respiratory tract infections in children and adults. Impaired mucociliary clearance with subsequent mucus retention contributes to the pathophysiology of each of these diseases, suggesting that altered epithelial salt and water transport may play an etiological role. To test the hypothesis that SHS would alter epithelial ion transport, we designed a system for *in vitro *exposure of mature, well-differentiated human bronchial epithelial cells to SHS. We show that SHS exposure inhibits cAMP-stimulated, bumetanide-sensitive anion secretion by 25 to 40% in a time-dependent fashion in these cells. Increasing the amount of carbon monoxide to 100 ppm from 5 ppm did not increase the amount of inhibition, and filtering SHS reduced inhibition significantly. It was determined that SHS inhibited cAMP-dependent apical membrane chloride conductance by 25% and Ba^2+^-sensitive basolateral membrane potassium conductance by 50%. These data confirm previous findings that cigarette smoke inhibits chloride secretion in a novel model of smoke exposure designed to mimic SHS exposure. They also extend previous findings to demonstrate an effect on basolateral K^+ ^conductance. Therefore, pharmacological agents that increase either apical membrane chloride conductance or basolateral membrane potassium conductance might be of therapeutic benefit in patients with diseases related to SHS exposure.

## Introduction

Tobacco use is a worldwide epidemic accounting for 3% of the world's morbidity and mortality at a cost of tens of billions of U.S. dollars annually [1]. Even in the United States of America, where smoking rates have declined over the last 4 decades, the prevalence of smoking among adults and teenagers remains approximately 22-24%, meaning that more than 66,000,000 people smoke regularly [1,2]. The wide prevalence of smoking means that many children and adults are exposed to SHS. A recent study released by the Social Climate Survey of Tobacco from the Mississippi State University http://socialclimate.org/ suggested that more than 40% of American children are exposed to secondhand smoke (SHS). This exposure is a significant risk factor for respiratory diseases, including lower airways infections, chronic rhinosinusitis, middle ear infection, and asthma in adults [3], as well as asthma and more severe respiratory syncytial virus (RSV) infection in children [4,5]. These diseases, while clearly multifactorial, all share a component of impaired mucociliary clearance (MCC) and mucus retention.

Maintenance of normal MCC in the respiratory tract depends on salt and water transport by respiratory epithelial cells. MCC is disrupted when epithelial salt and water transport is abnormal, as in cystic fibrosis (CF). Previous studies from our and others' laboratories demonstrated that components of cigarette smoke inhibited chloride (Cl^-^) secretion in polarized epithelia [6-8]. Mainstream cigarette smoke inhibited both CFTR expression and function both *in vitro *in immortalized cell lines and *in vivo *where nasal potential difference measurements were consistent with inhibition of Cl^- ^transport similar to that seen in cystic fibrosis [9]. These findings led us to hypothesize that SHS may have similar effects on epithelial Cl^- ^transport.

To test this hypothesis, we designed a system for *in vitro *exposure of mature, well-differentiated human bronchial epithelial cells (HBECs) to SHS. SHS (sometimes called environmental tobacco smoke or ETS) is a dilute combination of sidestream smoke released from burning cigarettes and a smaller amount of mainstream smoke exhaled by smokers. The components of SHS vary in concentration over time and distance from the source cigarette(s) [10,11]. A number of markers have been used to monitor the amount of SHS in the environment, including carbon monoxide (CO) and total suspended particulate (TSP) [12]. Though neither CO nor TSP is specific for SHS [13], they are easily measured and are correlated in non-ventilated conditions [14]. In households and cars, where most children are exposed to SHS, CO levels are in the range of 2.5-6 ppm [10,14], and thus we used this as the target exposure level in the majority of our experiments.

We measured baseline, amiloride-sensitive, cAMP-stimulated, and ATP-stimulated short-circuit current (I_SC_) in air (sham)-exposed and SHS-exposed HBECs. SHS inhibited forskolin-stimulated and ATP-stimulated I_SC_. Inhibition by SHS was not due to reduced cAMP production or protein kinase A (PKA) activity. Rather, SHS inhibited both apical CFTR conductance and basolateral, Ba^2+^-sensitive K^+ ^conductance, which provides the electrical driving force for Cl^- ^secretion in airway epithelia. These data support the hypothesis that SHS and primary cigarette smoke have similar effects on transepithelial ion transport in well-differentiated HBECs, and they suggest that therapies aimed at improving epithelial Cl^- ^secretion may be beneficial for people exposed to SHS.

## Materials and methods

### Cell culture

HBECs were purchased from Lonza (Walkersville, MD, USA), and cultured according to Gray and colleagues [15]. P2 HBECs were seeded into plastic T-75 flasks (Costar, Corning, Lowell, MA, USA) and grown in Bronchial Epithelial Cell Growth Medium (BEGM) (Lonza) supplemented with Ultroser-G (Pall Corporation, East Hills, NY, USA) (0.5% v/v), bovine pituitary extract (52 μg/mL), hydrocortisone (0.5 μg/mL), human recombinant EGF (0.5 ng/mL), epinephrine (0.5 μg/mL), transferrin (10 μg/mL), insulin (5 μg/mL), retinoic acid (0.1 μg/mL), triiodothyronine (6.5 μg/mL), gentamicin (50 μg/mL) and amphotericin-B (50 ng/mL). The medium was changed every 48 h until cells were 90% confluent. Cells were then collected and seeded at a density of 6 - 8 × 10^4 ^per 0.33 cm^2 ^onto Transwell permeable inserts (Costar) in differentiation media containing 50% DMEM in BEGM with the same supplements as above but lacking triiodothyronine and with a final retinoic acid concentration of 50 nM (all-trans retinoic acid; Sigma, ST. Louis, MO, USA). HBECs were maintained submerged for the first 7 d and then exposed to an apical air interface for the remainder of the culture period. The differentiation medium was refreshed 2 times each week. At all stages of culture, cells were maintained at 37°C in 5% CO_2 _in an air incubator. Under these conditions, HBECs formed a well-differentiated mucociliary phenotype with the classical ion transport phenotype associated with this tissue. HBECs were generally used between 4 and 8 weeks of age. We estimate that our cultures were 30 - 50% ciliated. A total of 7 donor cell lots were used to complete these studies and each experiment was performed using at least 2 different donor cell lots.

### Smoke exposure

**Figure 1:**
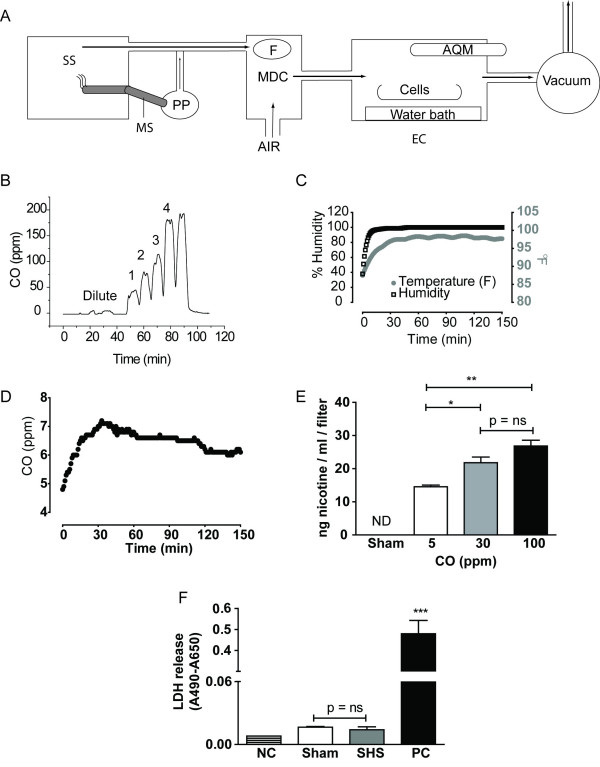
**Smoke Exposure System and quantification of exposure**. **A) **Secondhand smoke (SHS) was generated as described in the text. **B) **CO levels in the exposure chamber can be varied by diluting a single cigarette's smoke with fresh air (Dilute) or by smoking more than one cigarette simultaneously as indicated by the number of cigarettes shown above the tracing. Temperature and humidity **(C) **and carbon monoxide **(D) **are held constant after an initial equilibration period. **E) **Nicotine can be recovered from the mucosal surface of cells in the exposure chamber but not from sham-exposed cells. **F) **Sham and SHS-exposed (SHS) HBECs released LDH into the serosal exposure buffer to the same degree, and both released significantly less than cells permeabilized with 0.1% Triton-X100 as a positive control (PC) (*** p < 0.001 compared to both sham and SHS by one-way ANOVA with Tukey's post-tests, n = 4 inserts per condition). Exposure buffer not exposed to cells is shown as a negative control (NC) to demonstrate background.

SHS was generated using a custom-designed exposure system (Fig. 1A) similar to those used for *in vivo *SHS research [16]. Standardized research cigarettes (University of Kentucky, 1R5F) were ignited in an automated smoking machine (Teague TE-10, Teague Enterprises, Davis, CA, USA). Sidestream smoke was collected by negative pressure into a chimney above the burning cigarette. The TE-10 smoking machine puffer pump (PP) was programmed to take a 2 s, 35 mL puff of the burning cigarette every minute and mix the mainstream smoke (MS) with sidestream smoke (SS). Although the exact ratio of mainstream and sidestream smoke in the environment is unknown, this timing was designed to mimic a smoker inhaling and exhaling mainstream smoke once every minute. The mixture of sidestream and mainstream smoke, which we call whole cigarette smoke (WCS), was drawn into a mixing and dilution chamber (MDC) where it was mixed with room air by a fan (F). The dilute WCS, which we designate SHS, was then drawn at 5 L/min into a heated, humidified exposure chamber (EC) where the cells rested in HEPES-buffered solution (exposure buffer) on their serosal side and with their mucosal surface exposed. CO concentration in parts per million (ppm) was continuously monitored with a TSI Q-trak indoor air quality meter (AQM). When CO reached 4-5 ppm, the level of CO in SHS in households and cars, the inlet and outlet tubing connected to the exposure chamber were clamped with hemostats for the duration of the exposure. Sham (air) exposure was accomplished by placing cells with their apical surface exposed in a 37°C, 100% humidified, 0% CO_2 _incubator.

### Surface nicotine measurements by liquid chromatography/mass spectrometry

Liquid chromatography-mass spectrometry (LC-MS) was performed using a Waters Alliance 2690 HPLC system (Waters Corp., Milford, MA, USA). Gradient elution was performed in the linear mode using a Discovery HS F column (150 × 4 mm i.d., 5 mm; Sigma-Aldrich, St. Louis, MO) at a flow rate of 0.3 mL/min at ambient temperature.

MS was conducted with a Thermo Finnigan TSQ Quantum Ultra AM mass spectrometer (Thermo Fisher, San Jose, CA) equipped with a heated electrospray ionization (HESI) source operated in the positive ion mode. Unit resolution was maintained for both parent and product ions for multiple reaction monitoring (MRM) analyses. Operating conditions were as follows: spray voltage was 3500 V, vaporizer temperature was 300°C, and heated capillary temperature was 280°C. Nitrogen was used for the sheath gas and auxiliary gas set at 25 and 10 (in arbitrary units), respectively. Collision induced dissociation (CID) was performed using argon as the collision gas at 1.5 mTorr in the second (rf-only) quadrupole. An additional dc offset voltage was applied to the region of the second multipole ion guide (Q0) at 5 V. The MRM transition for nicotine was *m/z *163 to *m/z *84 (collision energy, 25 eV). Automated sample acquisition and data analysis were performed using the Xcalibur software (Thermo Scientific). Calibration curves were generated based on the peak-area ratios (analyte/internal standard) from 5 nM to 5 μM. Typical r^2 ^values were 0.995 or greater and the accuracy was between 90% and 100%.

### Short circuit current (I_SC_) measurements

Transwell inserts containing HBECs were mounted in a vertical Ussing chamber. HBEC monolayers were continuously clamped to 0 mV after fluid resistance compensation using an automatic voltage clamp (VCC 600, Physiologic Instruments, San Diego, CA, USA). I_SC _was digitized at 0.1 Hz, and data were stored on a computer hard drive using Acquire and Analyze software build 2.3.0 (Physiologic Instruments). Transepithelial resistance (R_T_) was determined automatically by the software using a bipolar 200 ms, 3 mV voltage pulse once per s, recording the change in I_SC_, and calculating R_T _from Ohm's law (R_T _= ΔV/ΔI). I_SC _was allowed to stabilize at the beginning of each experiment and after each drug addition. By convention, an upward deflection in the I_SC _tracing represents anion secretion or cation absorption.

### cAMP, PKA, and lactate dehydrogenase (LDH) measurements

Whole-cell cAMP levels and PKA activity, and LDH released by sham or SHS-exposed HBECs were measured using colorimetric assays according to manufacturer's instructions (Stressgen (Ann Arbor, MI, USA), Calbiochem (EMD Biosciences/Merck KGaA, Darmstadt, Germany), and Sigma, respectively). cAMP levels were measured in whole cell lysates collected after a 10 min incubation in 0.1 N HCl. cAMP levels were extrapolated from a standard curve are reported in pmol/mL. PKA levels were measured in whole cell lysates collected in a sample preparation buffer provided by the manufacturer. PKA activity is reported as absorbance units per microgram total protein used in the assay × 100. Total protein was determined by bicinchoninic acid assay (Sigma). LDH, a measure of release of intracellular components secondary to cytotoxicity, is reported as absorbance units.

### Solutions

During exposure (either sham or SHS) cells were bathed on the serosal side with a solution containing (in mM): 120 NaCl, 25 n-methyl-d-glucamine chloride (NMDG-Cl), 3.3 KH_2_PO_4_, 0.8 K_2_HPO_4_, 1.2 MgCl_2_, 1.2 CaCl_2_, 10 glucose and 10 HEPES, pH 7.4. This was done to minimize any possible effects of pH because no additional CO_2 _was added to the sham chamber or SHS exposure chamber. Ussing chamber solutions are detailed in Table 1.

**Table 1:** Ussing chamber solutions (in mM)

	Normal buffer	High Cl^-^	Low Cl^-^	High K^+^	Low K^+^
NaCl	120	145		25	25
NaHCO_3_^-^	25				
K_2_HPO_4_	0.8	0.8	0.8	0.8	0.8
KH_2_PO_4_	3.3	3.3	3.3	3.3	3.3
CaCl_2_	1.2	1.2	1.2	1.2	1.2
MgCl_2_	1.2	1.2	1.2	1.2	1.2
Na gluconate			145		120
K gluconate				120	
Glucose	10	10	10	10	10
HEPES		10	10	10	10
Gas lift	95% O_2_/5% CO_2_	Air	Air	Air	Air
pH		7.4	7.4	7.4	7.4

### Chemicals

Amiloride (Sigma) was dissolved in distilled, deionized water as 1000× stock. Forskolin (Calbiochem) was dissolved in DMSO as a 5000× stock. Bumetanide (Sigma) was dissolved in ethanol as a 1000× stock. The CFTR inhibitors glycine hydrazide-101 (GlyH-101) [17] and CFTRinh-172 [18], generous gifts of Dr. Robert J Bridges, Rosalind Franklin University and Cystic Fibrosis Foundation Therapeutics (CFFT), were dissolved in DMSO as 1000× stocks. 5,6-Dichloro-1-ethyl-1,3-dihydro-2H-benzimidazol-2-one (DC-EBIO) (Tocris, Ellisville, MO, USA) was dissolved in DMSO as a 1000× stock.

### Data Presentation and Statistical analysis

We report I_SC _data in two ways. In the text, we report mean I_SC _+/- standard error for the total number of inserts used to complete each experiment. We observed significant donor-to-donor and lot-to-lot variability in forskolin-stimulated I_SC_. For example, in our initial group of experiments examining acute exposure to SHS with 5 ppm CO the inter-donor forskolin-stimulated I_SC _ranged from 0.6 μA/cm^2 ^to 29 μA/cm^2^. In the same experiments, the intra-donor variability in forskolin-stimulated I_SC _was as high as 50%. To account for this variability, data were normalized by calculating the mean change in I_SC _for the sham-exposed HBECs from a given donor on the day of experimentation and normalizing each sham and SHS-exposed insert tested on that day by this result. Because we were interested in the relative change in I_SC _with SHS exposure, statistical comparisons were performed on the normalized data. Bar graphs of normalized data are presented so that the direction of the bar is consistent with the direction of change in I_SC_. Comparisons between groups with equal variances were made with unpaired *t*-tests, and comparisons between groups with unequal variances were made with unpaired *t*-tests using Welch's correction. All statistical comparisons were made using Prism 5 (GraphPad Software, San Diego, CA). Significance was defined as a p value = 0.05.

## Results

### Quantification of CO and nicotine exposure

CO concentration in the exposure chamber was used as a surrogate marker for SHS exposure and was measured using a TSI Q-Trak air quality monitor (TSI Inc., Shoreview, MN). Depending on the number of cigarettes simultaneously smoked and the amount of fresh air drawn into the mixing and dilution chamber, the system generated between 0 and 200 ppm CO (Fig. 1B). Temperature and humidity were maintained at physiological values (Fig. 1C). By clamping the inlet and outlet tracts of the exposure chamber, CO concentration could be held within a narrow range (5 - 10 ppm) (Fig. 1D), although there was a slow drift of CO. To demonstrate deposition of a known constituent of cigarette smoke onto cells in the chamber, we placed 200 μl of warm PBS on the apical surface of HBECs exposed to SHS for 30 min and measured nicotine in the collected mucosal surface washes by mass spectrometry. Nicotine was not detected in sham-exposed HBECs. Nicotine was detected in all samples exposed to SHS, and there was more nicotine deposited on HBECs exposed to 30 and 100 ppm CO compared with 5 ppm (n = 6 inserts for 5 ppm, 4 inserts for 30 ppm, and 4 inserts for 100 ppm, p < 0.001 for both by one-way ANOVA with Tukey's post-tests), but there was no statistically significant difference between 30 and 100 ppm (Fig. 1E).

### Exposure to SHS did not change R_T _or increase LDH release

To determine if SHS caused cellular toxicity that impaired membrane integrity, we measured R_T _as well as LDH release following SHS exposure. HBECs were exposed to either air (sham) or SHS for 180 min (the longest exposure time used in subsequent experiments). Immediately following exposure, the Transwell inserts were mounted in Ussing chambers and the transepithelial potential was clamped to 0 mV. Baseline R_T _was not different between sham and SHS-exposed HBECs after 180 min exposure (936 ± 86 Ωcm^2 ^vs. 926 ± 63 Ωcm^2^, respectively, n = 19 inserts, 3 donors, p = 0.9). In addition, there was no difference in the amount of LDH released into the exposure medium of sham versus SHS-exposed cells (n = 4 inserts for each condition, p = 0.4). As a control, significant LDH release was detected in cells that had their mucosal surface exposed to 0.1% Triton-X100 (Fig. 1F, p < 0.001 by one-way ANOVA). Together, these data suggest that acute SHS exposure does not cause a generalized disruption of epithelial integrity. However, it should be noted that LDH release into exposure media measures primarily basolateral membrane integrity, whereas the apical membrane of the cells received the direct exposure.

### SHS inhibits cAMP and ATP-stimulated Cl^- ^secretion

**Figure 2:**
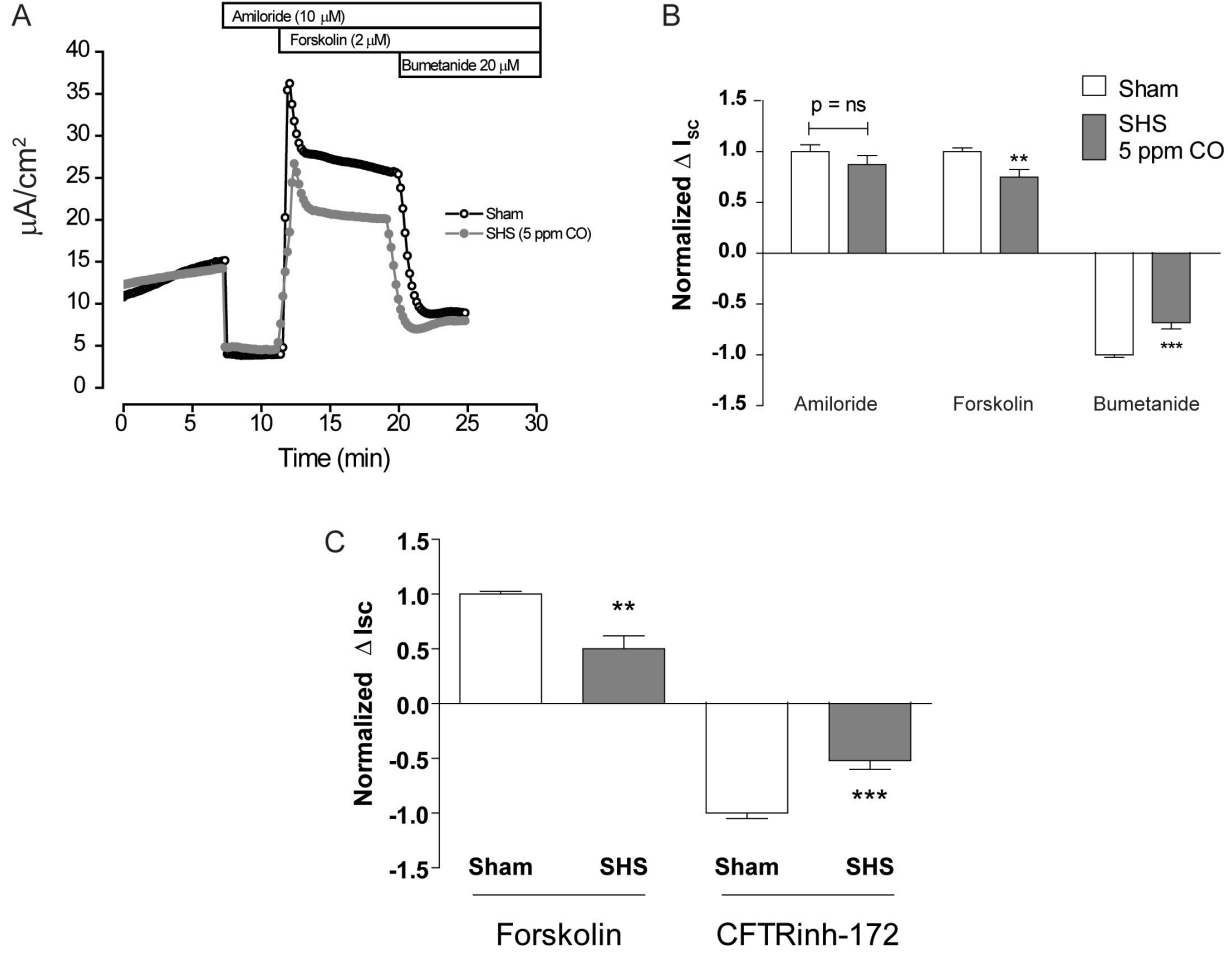
**SHS inhibits forskolin-stimulated Cl^- ^secretion**. **A) **Representative I_SC _tracing after 30 min sham (open circles) or SHS exposure (gray circles). Note that the decrease in forskolin-stimulated I_SC _in SHS-exposed HBECs is completely accounted for by a decrease in bumetanide sensitive I_SC_. **B) **Normalized changes in I_SC _with amiloride, forskolin, and bumetanide (** p < 0.01 and *** p < 0.001 by unpaired t-test, n = 32 inserts, 5 donors). **C) **Normalized changes in I_SC _with forskolin and CFTRinh-172 (** p < 0.01 and *** p < 0.001 by unpaired t-test, n = 6 filters, 2 donors).

To determine the acute effect of SHS on transepithelial ion transport, HBECs were exposed to air (sham) or SHS (5 - 10 ppm CO) for 30 min and then immediately mounted in Ussing chambers. Baseline I_SC _and R_T _were recorded prior to the sequential addition of amiloride, forskolin, and bumetanide (Fig. 2A). Baseline I_SC _and R_T _were not different between sham and SHS-exposed HBECs (Baseline I_SC_: 11.5 ± 1.0 for sham vs. 10.4 ± 1.0 μA/cm^2 ^for SHS, n = 32 inserts, 5 donors, p = ns; Baseline R_T_: 1261 ± 145 for sham vs. 1429 ± 162 Ωcm^2 ^for SHS, p = 0.44). Similarly, amiloride-sensitive I_SC _was not different between sham and SHS-exposed cells, suggesting that acute SHS exposure did not affect ENaC-mediated Na^+ ^absorption (Fig. 2B, left bars). In contrast, SHS exposure (30 min, 5 ppm CO) reduced forskolin-stimulated I_SC _by 25% (14.2 ± 1.3 μA/cm^2 ^for sham vs. 9.7 ± 1.0 μA/cm^2 ^for SHS, n = 32 inserts, 5 donors, p < 0.01) (Fig. 2B, middle bars). This reduction in forskolin-stimulated I_SC _was paralleled by a reduction in bumetanide-sensitive I_SC _(Fig. 2B, right bars), suggesting that SHS specifically reduced Cl^- ^secretion [19,20]. To investigate if the reduction in forskolin-stimulated I_SC _was dependent on CFTR, a similar experiment was performed in which HBECs were sham or SHS-exposed for 10 min, mounted in Ussing chambers, and sequentially exposed to amiloride, forskolin, and CFTRinh-172. Similar to the results seen with bumetanide, CFTRinh172-sensitive I_SC _was reduced to the same extent as forskolin-stimulated I_SC _(Fig. 2C), strongly suggesting that SHS inhibits CFTR-dependent Cl^- ^secretion.

To investigate the effect of SHS on Ca^2+^-activated Cl^- ^secretion, HBECs were either sham or SHS-exposed for 180 min (the longest duration of exposure used in the cAMP experiments, see below) and then immediately mounted in Ussing chambers. Amiloride and then ATP (200 μM) were sequentially added to the mucosal bath. Addition of ATP resulted in a large peak in I_SC _followed by a plateau. After 180 min of SHS peak ATP-stimulated I_SC _was inhibited by 40% (33.1 ± 5.2 μA/cm^2 ^for sham vs. 18.4 ± 2.0 μA/cm^2 ^for SHS, n = 7 inserts, 2 donors, p = 0.02 by unpaired t-test) whereas plateau I_SC _was inhibited by 30% (11.4 ± 1.1 mA/cm2 for sham vs. 8.1 ± 1.0 mA/cm2 for SHS, p = 0.04 by unpaired t-test).

### The effect of SHS on forskolin-stimulated Cl^- ^secretion is time-dependent and not reversible up to 24 hrs

**Figure 3:**
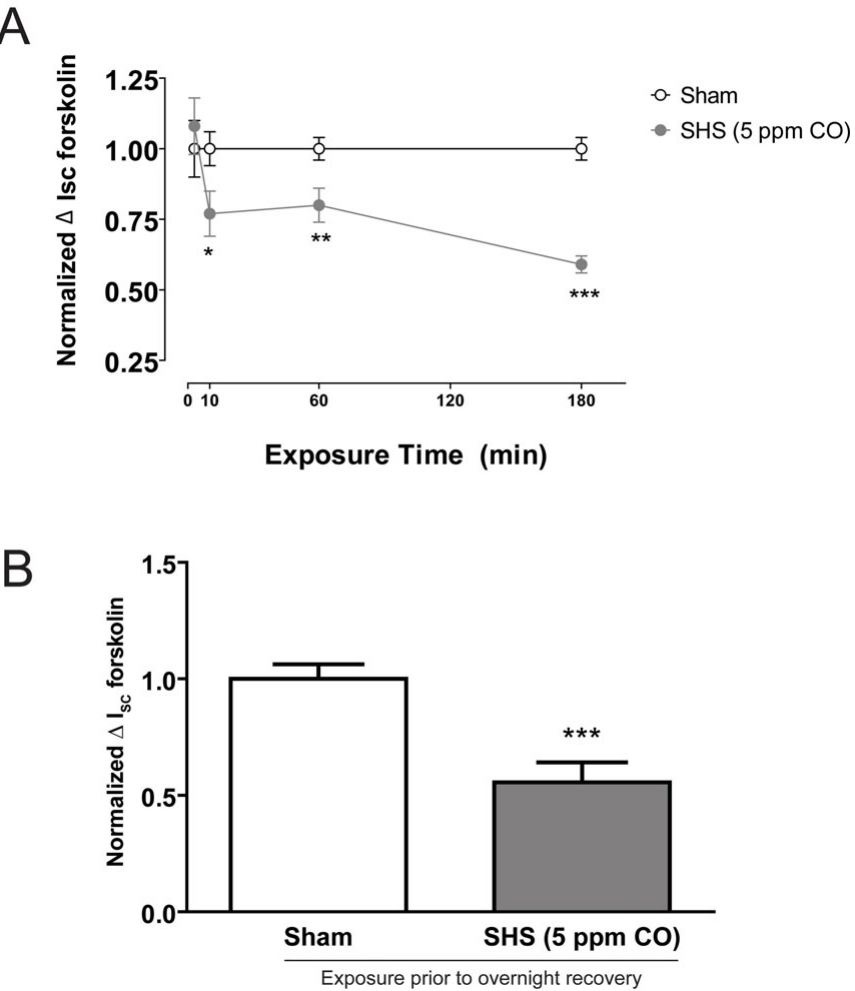
**Inhibition of forskolin-stimulated Cl^- ^secretion is time-dependent**. **A) **Normalized change in I_SC _after forskolin stimulation is similar between sham (open circles) and SHS-exposed (shaded circles) HBECs at 3 min, but decreased at 10 and 60 min (* p ≤ 0.05 by unpaired t-test, n = 8 inserts, 2 donors and 17 inserts, 3 donors, respectively). Change in forskolin-stimulated I_SC _is further decreased in the SHS-exposed group at 180 min (*** p ≤ 0.001 by unpaired t-test, n = 19 inserts, 3 donors). Normalized I_SC _for sham-exposed HBECs remains 1 because each time-point is normalized independently. **B) **Normalized change in I_SC _after forskolin stimulation is still decreased after an overnight recovery period (*** p ≤ 0.001 by unpaired t-test, n = 12 inserts, 3 donors).

Our previous data using water-soluble cigarette smoke extract (CSE) suggested that water-soluble components of cigarette smoke inhibited forskolin-stimulated I_SC _after as little as 5 min, but that maximal inhibition was not achieved for approximately 30 min [6]. After 3 min of SHS exposure, forskolin-stimulated I_SC _was not different between SHS-exposed HBECs and sham controls (9.5 ± 1.5 μA/cm^2 ^vs. 9.2 ± 0.8 μA/cm^2^, respectively, n = 13 inserts, 3 donors, p = 0.87) (Fig. 3A). After 10 min or 60 min, forskolin-stimulated I_SC _was reduced by approximately 20%, a trend that did not reach significance for the raw data but did for the normalized data (10 min: 17.5 ± 2.2 μA/cm^2 ^for sham vs. 13.1 ± 1.4 μA/cm^2 ^for SHS, n = 8 inserts, 2 donors, p = 0.15; 60 min: 13.7 ± 1.0 μA/cm^2 ^for sham vs. 11 ± 1.0 μA/cm^2 ^for SHS, n = 17 inserts, 3 donors, p = 0.07). After 180 min, forskolin-stimulated Cl^- ^secretion was decreased by 40% in SHS-exposed HBECs compared to sham controls (18.2 ± 1.8 μA/cm^2 ^for sham 10.7 ± 1.3 μA/cm^2 ^for SHS, n = 19 inserts from 3 donors, p < 0.01). These data suggest that inhibition of Cl^- ^secretion in HBECs by SHS is time-dependent.

We next investigated whether inhibition of Cl^- ^secretion by SHS was reversible. HBECs were exposed to air or SHS (5 - 10 ppm CO) for 180 min then the mucosal surface was rinsed with warm PBS three times prior to an overnight recovery period with the inserts bathed on the serosal side in fresh medium in a new tissue culture plate. Even after an overnight recovery period forskolin-stimulated Cl^- ^secretion in the SHS-exposed HBECs was still decreased compared to sham controls (Fig. 3B).

### Inhibition of forskolin-stimulated Cl^- ^secretion is not dependent on CO concentration and filtering SHS reduces its impact

**Figure 4:**
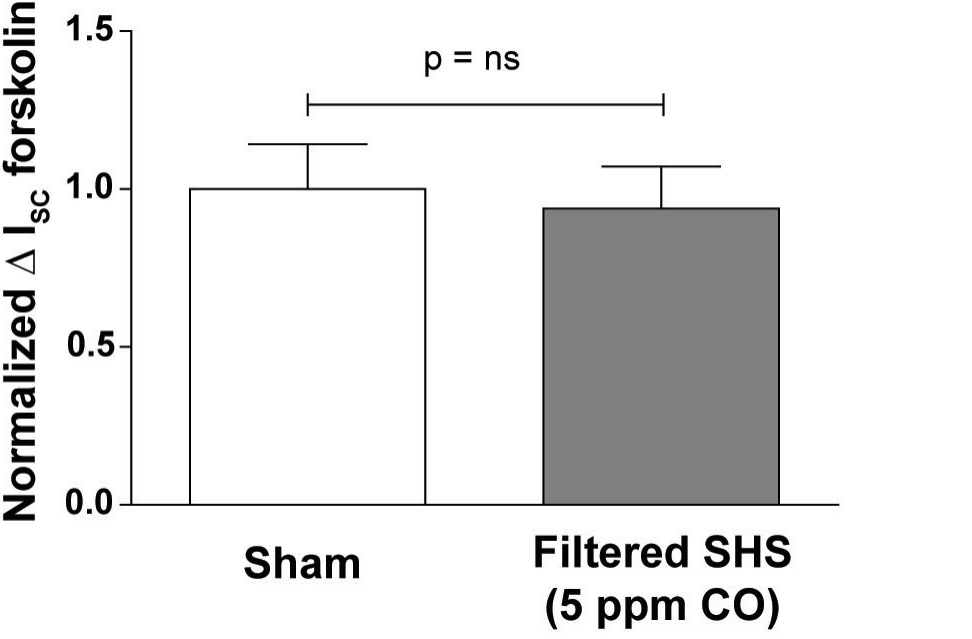
**Filtered SHS does not reduce forskolin-stimulated Cl^- ^secretion**. A 2 μm Teflo air sampling filter (Pall Corporation) was placed over the inlet port of the exposure chamber. With the filter in place, 180 min SHS exposure (5 ppm CO) had no effect on forskolin-stimulated Cl^- ^secretion.

To examine the relationship between amount of exposure as measured by CO and inhibition of Cl^- ^secretion we increased the exposure chamber CO concentration to 100 ppm from 5 ppm. SHS with 100 ppm CO, a level above that which one would expect with environmental SHS exposure, appeared to inhibit forskolin-stimulated I_SC _(13 ± 1.6 μA/cm^2 ^for sham vs. 9.6 ± 1.3 μA/cm^2 ^for SHS, n = 11 inserts, 3 donors), though the difference did not reach statistical significance when analyzing either the raw data or the normalized data (p = 0.1 and 0.3, respectively). The lack of a direct relationship between CO concentration and the degree of Cl^- ^secretion inhibition by SHS suggested the hypothesis that the particulate phase rather than the gaseous phase of the smoke was responsible for the effect [7] because CO resides in the gaseous phase of SHS. To test this, we sealed the inlet to the exposure chamber with a 2.0 micrometer Teflo filter (Pall, Inc.) to trap particulate, but not gaseous SHS constituents. With the filter in place CO concentration inside the exposure chamber could still be modulated, but a 180 min SHS exposure (5-10 ppm CO) did not significantly reduce forskolin-stimulated Cl^- ^secretion (Fig. 4, n = 8 inserts, 2 donors). These data support the hypothesis that a filterable, perhaps particulate component of SHS causes inhibition of Cl^- ^secretion.

### SHS does not affect cAMP production or PKA activity

**Figure 5:**
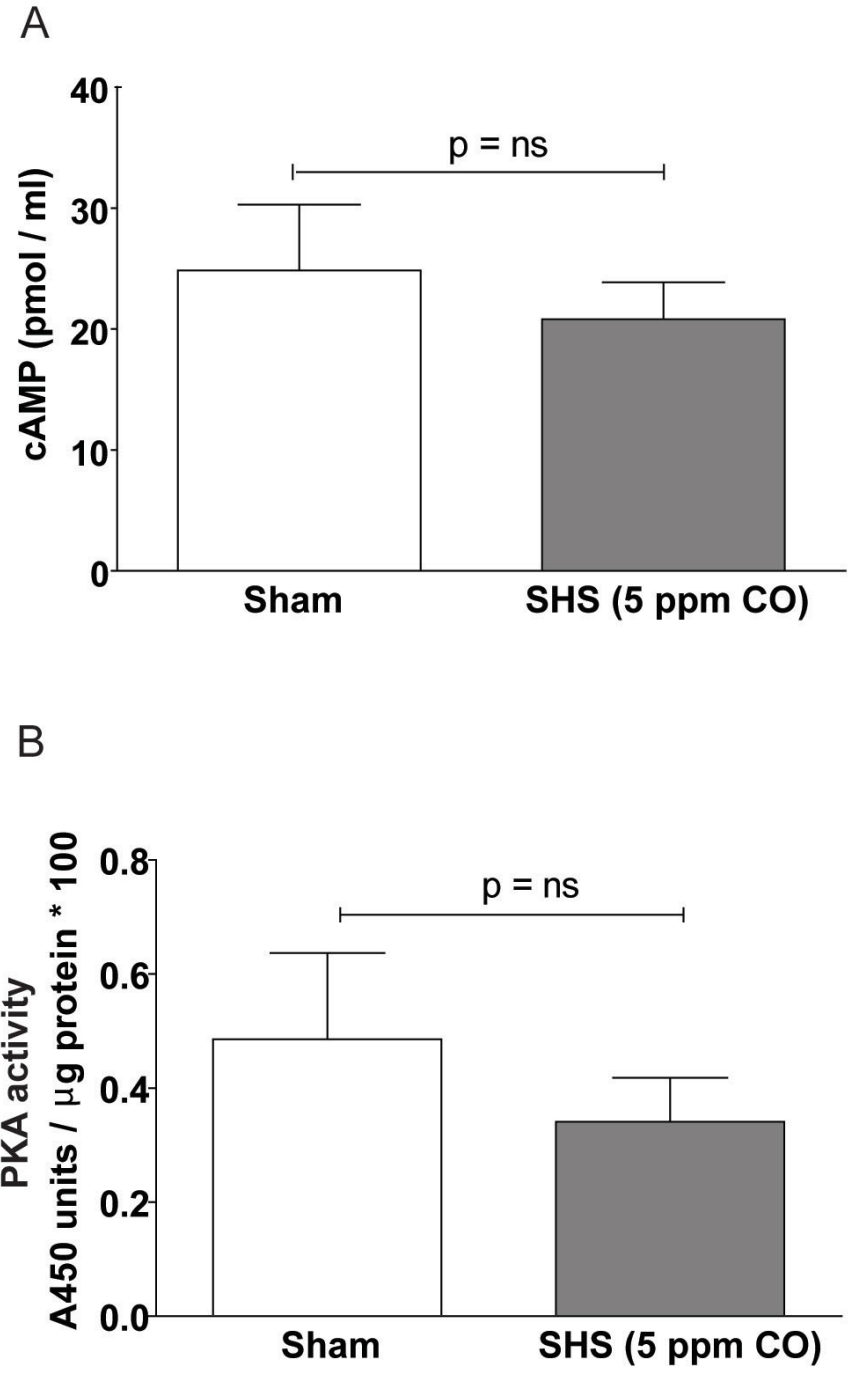
**SHS does not appear to affect cAMP production or PKA activity**. Whole-cell cAMP (A) and PKA activity (B) were measured as described in Materials and Methods. There was no statistically significant difference in cAMP production or PKA activity between sham and SHS-exposed HBECs.

Forskolin activates Cl^- ^secretion in HBECs by stimulating transmembrane adenylate cyclase (tm-AC) to increase cellular cAMP levels. cAMP then binds to PKA causing release of catalytic PKA subunits that phosphorylate CFTR. Therefore, we performed experiments to determine if SHS inhibited Cl^- ^secretion by interrupting this signaling cascade. HBE cells were exposed to air or SHS (5 ppm CO, 30 min) and then stimulated with forskolin after which total cell lysates were collected. Forskolin stimulation elevated cAMP levels equivalently in both the sham and SHS-exposed cells, suggesting that the SHS-mediated inhibition of Cl^- ^secretion is not due to a defect in tm-AC activity (Fig. 5A, n = 4 inserts for sham and 3 inserts for SHS, p = 0.6. Note: 1 insert exposed to SHS was an outlier with a cAMP level > 200 pmol/ml). Using a similar experimental design, there was no statistical difference in whole-cell PKA activity between sham and SHS-exposed cells, though there was a trend toward less PKA activity in SHS-exposed HBECs (Fig. 5B, n = 12 inserts, 3 donors, p = 0.4 by unpaired t-test with Welch's correction).

### Acute exposure to SHS inhibits both apical Cl^- ^conductance and basolateral K^+ ^conductance

Because SHS did not appear to affect cAMP signaling, we next investigated the effects of SHS on cAMP-dependent ion conductances. cAMP-stimulated Cl^- ^secretion depends on activation of both apical membrane CFTR [21] and basolateral membrane K^+ ^channels [22]. Therefore, we investigated the effect of SHS on both.

**Figure 6:**
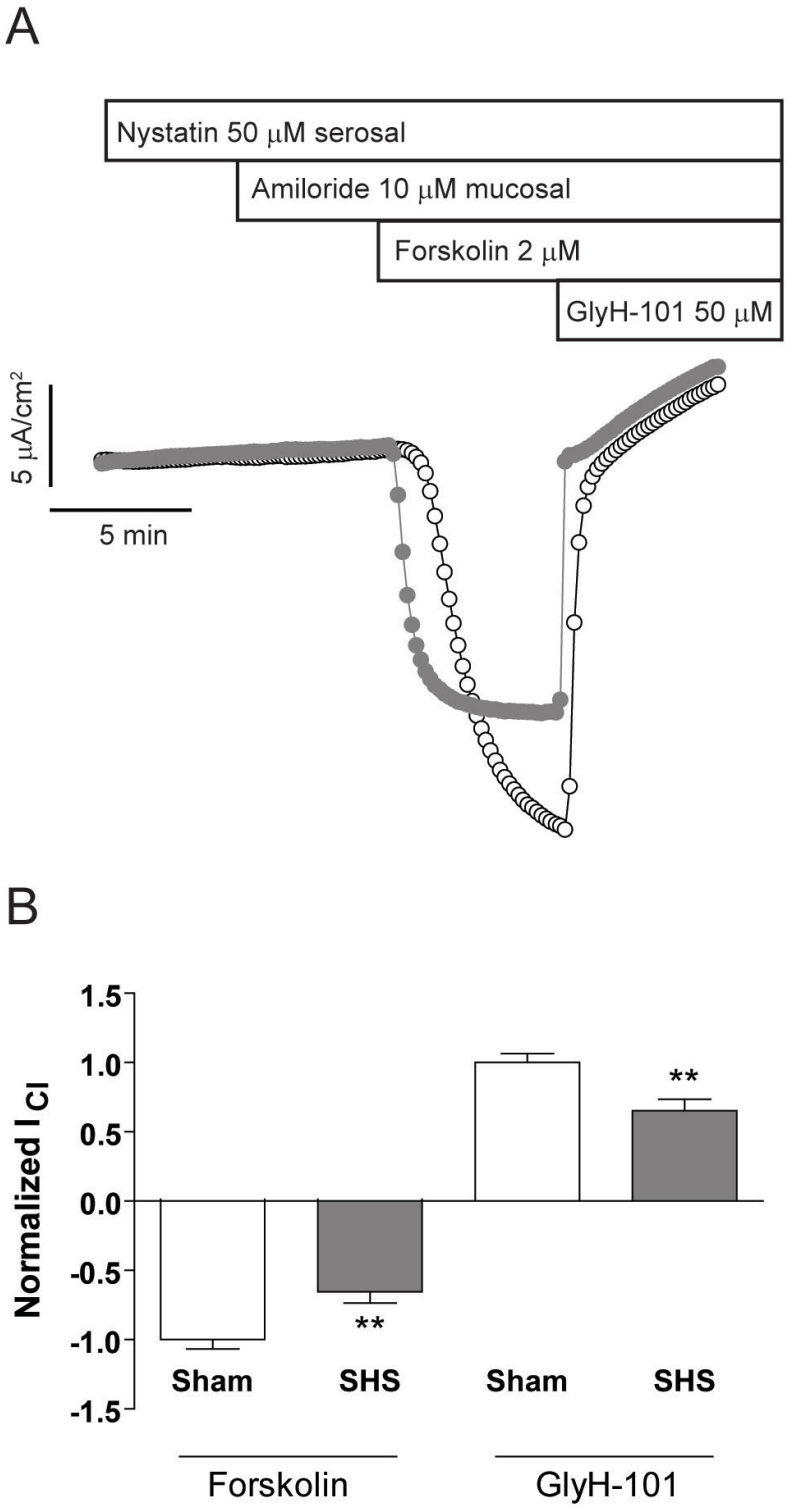
**SHS reduces apical membrane Cl^- ^conductance**. **A) **Representative current tracings (sham: open circles; SHS: shaded circles) demonstrating that nystatin-permeabilization of the basolateral membrane allows for a diffusive Cl current (I_Cl_) that can be inhibited by GlyH-101, a blocker of CFTR channels. Note that addition of amiloride has no effect on current, confirming that the intracellular Na^+ ^concentration was defined by the serosal bath solution. **B) **Normalized changes in I_Cl _with forskolin and GlyH-101 (** p ≤ 0.01 by unpaired t-test).

To test the hypothesis that SHS inhibited Cl^- ^secretion by decreasing apical membrane Cl^- ^conductance, we performed Ussing chamber studies in which the basolateral membrane was permeabilized with nystatin [23]. Sham or SHS-exposed HBECs were mounted in Ussing chambers in the presence of a mucosal-to-serosal Cl^- ^gradient (30:1) and then the basolateral membrane was permeabilized with 50 μM nystatin. Permeabilization was confirmed by the inability of amiloride to reduce I_SC_. Application of forskolin resulted in a diffusive Cl^- ^current (I_Cl_) seen as a downward deflection in the current tracing, that could be inhibited by the CFTR blocker GlyH-101 (Fig. 6A). These studies indicated that SHS exposure inhibited 35% of forskolin-stimulated I_Cl _(-19.1 ± 2.6 μA/cm^2 ^for sham vs. -11.8 ± 1.8 μA/cm^2 ^for SHS, p = 0.03) and GlyH-101-sensitive I_Cl _(23.2 ± 3.6 μA/cm^2 ^for sham vs. 14.9 ± 2.8 μA/cm^2 ^for SHS, n = 14 inserts, 3 donors, p = 0.08) (Fig 6B).

**Figure 7:**
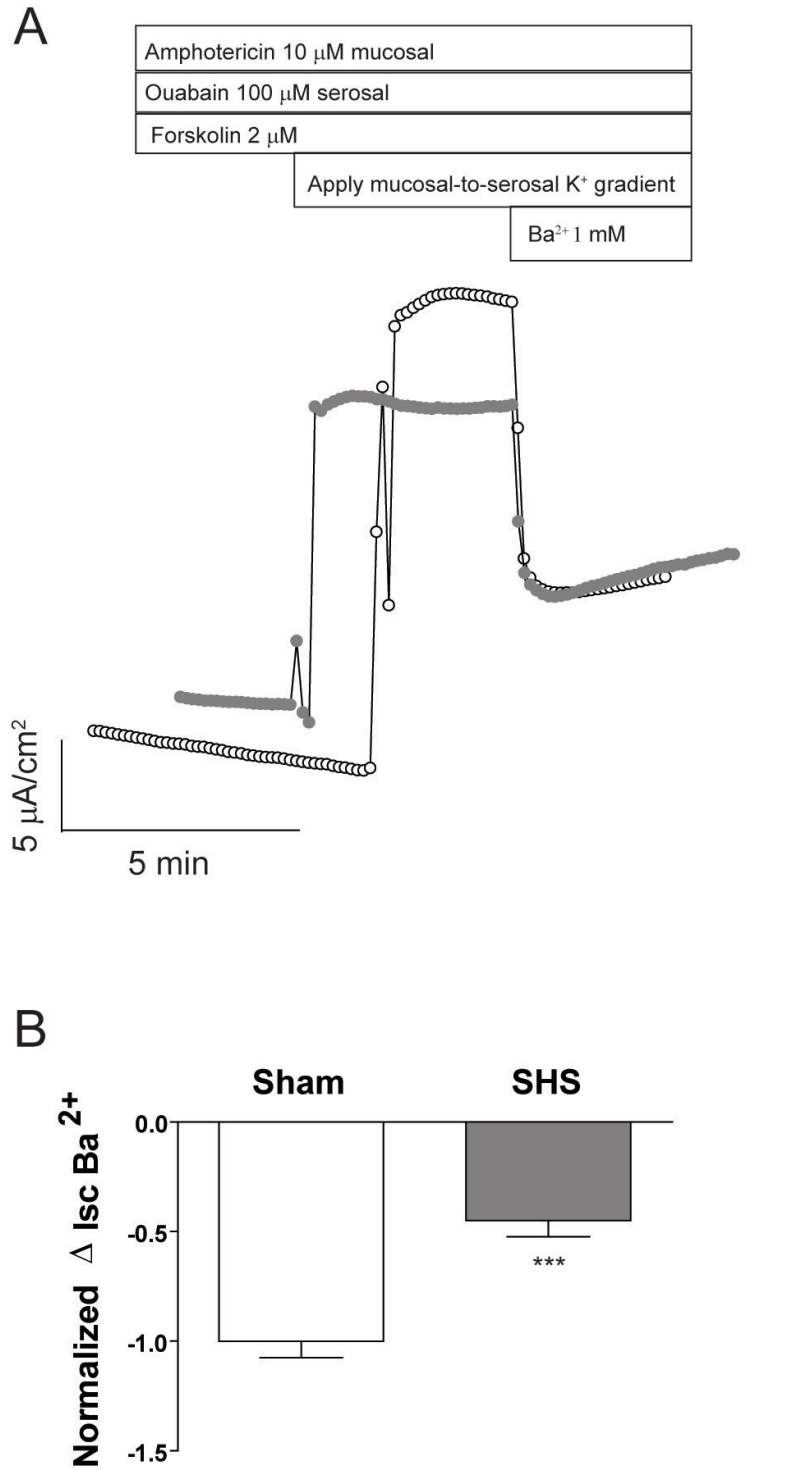
**SHS reduces basolateral membrane K^+ ^conductance**. **A) **Representative tracings (sham: open circles; SHS: shaded circles) demonstrating that amphotericin-permeabilization of the apical membrane allows for a diffusive K current (I_K_) that can be inhibited by Ba^2+^, a blocker of many K^+ ^channels. **B) **Normalized changes in I_K _after sequential application of a K^+ ^gradient and Ba^2+ ^(*** p ≤ 0.001 by unpaired t-test).

We next investigated the effects of SHS exposure on basolateral K^+ ^conductance. In these experiments, sham or SHS-exposed HBE cells were initially bathed in symmetrical high K^+ ^solutions and then the apical membrane was permeabilized with 10 μM amphotericin B [24] in the presence of 100 μM ouabain (to inhibit Na^+^/K^+ ^ATPase activity) and 2 μM forskolin (to activate tm-AC). A mucosal-to-serosal K^+ ^gradient (25:1) was established by exchanging approximately 20 volumes (60 ml into a 3 ml Ussing chamber volume) of the serosal bath for a low K^+ ^solution. A diffusive K^+ ^current (I_K_) was seen as an upward deflection in the current tracing that peaked to varying degrees and then fell to a stable plateau. The resulting steady-state I_K _was inhibited by Ba^2+ ^(Fig. 7A). Because the peak occurred during the solution exchange and was variable both in timing and amplitude, we used the Ba^2+^-sensitive change in I_K _as our measurement of basolateral membrane K^+ ^conductance. SHS exposure reduced basolateral K^+ ^conductance by 50% (Fig. 7B). Taken together, our data suggest that SHS-reduced epithelial Cl^- ^secretion involves inhibition of both apical membrane CFTR and basolateral K^+ ^channels.

## Discussion

We investigated the effects of SHS on airway epithelial cell ion transport using a system that approximates *in vivo *SHS exposure and is similar in design to ones used for *in vivo *SHS toxicology [16,25] and *in vitro *primary smoke exposure [26,27]. Environmental monitoring suggests that SHS raises the CO concentration of indoor rooms and car interiors to approximately 2.5 - 6 ppm [10,14]. Our data demonstrate that it is feasible to expose HBECs at physiological temperature and humidity to this level of SHS without causing significant generalized disruption of epithelial integrity, as demonstrated by lack of effects on R_T _and LDH release. A limitation of this system is that some components of SHS may be lost as smoke passes through the exposure apparatus. During SHS exposure, HBECs in our system are exposed to approximately 45 ng nicotine/ml/cm^2 ^(Fig. 1E) when CO is 5 ppm. This is equivalent to a nicotine concentration of 270 nM, approximately 120× lower than the 33 μM measured in the expectorated sputum of smokers immediately following smoking of a single cigarette [27]. To our knowledge, the amount of nicotine found in the airways of children exposed to SHS is unknown, but it is likely that it is significantly less than that found in smokers because of the dilution and aging of SHS in room air.

SHS exposure is associated with diseases that are also associated with impaired MCC, including asthma, chronic rhinosinusitis, and lower airways infections [3,4,28], but the cellular mechanisms by which SHS might impair MCC remain incompletely characterized. One hypothesis is that SHS impairs epithelial ion transport because abnormal epithelial ion transport can negatively affect MCC [29]. For example, in cystic fibrosis (CF) the near total absence or dysfunction of CFTR, the primary cAMP-dependent Cl^- ^channel in airway epithelial cells, causes altered salt and water transport by both surface epithelial cells [30,31] and submucosal glands [32] that results in impaired MCC and chronic mucopurulent sinusitis and bronchitis. Furthermore, even partial decreases in CFTR expression or function may have clinical implications. Epidemiological data suggest that heterozygote carriers of CFTR mutations (who do not have CF) have an increased prevalence of asthma [33] and chronic rhinosinusitis [34] compared to the general population, and there is a higher prevalence of CFTR missense mutations in people with asthma than in the general population [35]. Therefore, intermediately decreased CFTR function, as observed with SHS exposure in the present study, may result in clinically significant changes in MCC in some circumstances.

Some evidence suggests that cigarette smoke inhibits CFTR-dependent Cl^- ^transport *in vivo *[9], and we and others have demonstrated that water-soluble components of cigarette smoke inhibit Cl^- ^secretion *in vitro *[6,7]. A limitation of these studies was the reliance on extracts of cigarette smoke that may or may not accurately represent the chemical composition of whole (or gaseous) cigarette smoke [27,36]. We report here that exposure of the mucosal surface of well-differentiated HBECs to SHS did not affect amiloride-sensitive Na+ absorption but inhibited forskolin-stimulated and ATP-stimulated I_SC_, measures of the epithelial anion (Cl^-^, HCO_3_^-^) secretion that drives fluid secretion. Using radioisotopic Cl^- ^flux measurements, we previously demonstrated that CSE specifically inhibited Cl^- ^secretion [6]. Our studies here demonstrated that the decrease in forskolin-stimulated I_SC _was completely accounted for by a decrease in bumetanide-sensitive I_SC _(Fig. 2B, right) or a decrease in CFTRinh-172-sensitive I_SC_, agreeing with our previous data and suggesting that SHS acts similarly to CSE with respect to inhibition of CFTR-dependent Cl^- ^secretion.

Our data do not directly identify the compound (or compounds) in SHS that inhibit Cl^- ^secretion in HBECs. CO is an unlikely candidate because it did not correlate with the degree of inhibition of Cl^- ^secretion. Interestingly, the relative decrease in forskolin-stimulated I_SC _by SHS with 100 ppm CO was similar to that of 5 ppm CO but did not reach statistical significance, suggesting that higher concentrations of CO or another gaseous component of SHS might have an effect on Cl^- ^secretion that is not addressed by these studies. A particulate component of SHS is a more likely candidate as we found that the particulate phase of SHS is necessary for inhibition of Cl^- ^secretion (Fig. 4), complementing previous data that the particulate phase of cigarette smoke is sufficient for inhibition of Cl^- ^secretion [6,7]. Taken together these data strongly implicate a component of the particulate phase of SHS as the inhibiting agent.

There are multiple ways by which SHS may inhibit forskolin-stimulated Cl^- ^secretion. First, SHS may interfere with forskolin-activated tm-AC activity or downstream PKA activity. While our data (Fig. 5) show a trend toward inhibition of PKA activity, they did not achieve statistical significance. Furthermore, we measured only whole-cell cAMP levels and PKA activity; therefore, we cannot exclude the possibility that SHS alters cAMP levels or PKA activity in critical subcellular domains [37] and so do not completely discount the possibility that SHS impairs cAMP signaling.

Second, SHS may inhibit the ion channels that are involved in transepithelial Cl^- ^secretion. Previous data suggest that mainstream cigarette smoke reduces whole-cell CFTR expression and function in non-polarized Calu-3 cells [9]. In agreement with these findings, a 30 min SHS exposure inhibited approximately 25% of forskolin-stimulated and GlyH-101-sensitive I_Cl _in permeabilized HBEC monolayers (Fig. 6). One possibility to explain the decrease in CFTR conductance is that SHS contains a compound (or compounds) that rapidly decreases the open probability (P_O_) of CFTR as has been shown for oxidized forms of glutathione [38]. However, Cd^2+^, a prominent constituent of cigarette smoke, rapidly increases opening of CFTR channels [39], so it is likely that if there are effects of SHS on channel gating that they will be complex and difficult to tease out. Furthermore, SHS exposure maximally inhibited Cl^- ^secretion by 180 min but had no effect after 3 min (Fig. 3A), though there was some variability in the time-dependence of these responses that may have been due to difference in cell lot variability or differences in toxin deposition in the exposure chamber. The time frame for inhibition of forskolin-stimulated I_SC _was therefore longer than expected for direct channel blockade. Also, the inhibition of Cl^- ^secretion was not reversible by washing the mucosal surface of the cells and allowing them to recover overnight (Fig. 3B), which may be because the cells or the support on which they are grown retained some particulate. Because the effect of SHS was time-dependent and not reversed by washing of the apical membrane, we speculate that SHS exposure acutely affects either or both transcriptional and post-transcription modulation of CFTR expression and trafficking. This mechanism would be consistent with the previously reported decrease in total cellular CFTR [9].

In addition to inhibiting apical membrane Cl^- ^conductance, SHS also inhibited 50% of Ba^2+^-sensitive, basolateral membrane K^+ ^conductance (Fig. 7). This finding suggests that Na^+ ^absorption, which was not affected by SHS, is not entirely dependent on Ba^2+^-sensitive K^+ ^channels. Rather, there may be non-Ba^2+^-sensitive K^+ ^channels that help to maintain membrane potential favorable for Na^+ ^absorption even in the presence of SHS. These conductances may be down-regulated after amiloride block [40], so that they are absent when examining Cl^- ^secretion in the presence of amiloride as we have done in these studies.

We can speculate on a number of possible explanations for the observed decrease in K^+ ^conductance. First, there are numerous heavy metals in cigarette smoke that are pore blockers of K^+ ^channels. Cd^2+^, for example, inhibits KCNQ1 channels [41], which participate in transepithelial Cl^- ^secretion in HBECs [42]. Second, membrane conductances can be coordinated in epithelial cells [43], so the decrease in K^+ ^conductance may be a response to the reduction in apical membrane Cl^- ^conductance, similar to the response seen with reduction in apical membrane Na^+ ^conductance [40]. Alternatively, we cannot exclude the possibility that the primary effect of SHS on Cl^- ^secretion is decreased basolateral membrane K^+ ^conductance and that apical membrane Cl^- ^conductance is decreased as a compensatory response.

Many acute toxicological effects of cigarette smoke are attributable to oxidative stress [44]. Data regarding the effects of oxidative stress on CFTR-mediated Cl^- ^secretion are conflicting, with reports of oxidative stress causing both increased [45] and decreased [46] Cl^- ^secretion. These differences may be explained by differences in cell culture model systems, the type of oxidant used to generate oxidative stress, which will generate different types of oxygen radicals, or differences in the amount of oxidative stress that was induced.

There are data to support redox-dependent regulation of both CFTR [38,39,47] and K^+ ^channels including KCNQ1 [48] (reviewed in [49]). Therefore, hypothesizing that oxidative stress caused by SHS results in inhibition of Cl^- ^secretion is reasonable, particularly because oxidative stress has been shown to decrease CFTR expression in epithelial cells [50]. However, it is important to point out that previous investigators were unable to reverse the effects of cigarette smoke on epithelial Cl^- ^secretion with antioxidants [7]. Given the thousands of biologically active compounds in cigarette smoke, further investigations will be necessary to delineate the molecular mechanism of the observed inhibition.

Although we do not know the mechanism by which ion conductances are decreased, we do know that pharmacological agents that promote Cl^- ^secretion, such as activators of Ca^2+^-activated Cl^- ^secretion, improve lung function in patients with CF [51]. Similarly, pharmacological agents that increase either apical membrane Cl^- ^conductance or basolateral membrane K^+ ^conductance to promote Cl^- ^secretion in airway epithelial cells might be of clinical benefit in respiratory diseases caused by SHS exposure or smoking [52].

## Abbreviations

(SHS): Secondhand smoke; (MCC): mucociliary clearance; (HBECs): human bronchial epithelial cells; (CO): carbon monoxide; (CF): in cystic fibrosis; (TSP): total suspended particulate; (PKA): protein kinase A; (cAMP): 3'-5'-cyclic adenosine monophosphate; (I_SC_): short-circuit current; (R_T_): transepithelial resistance; (CFTR): cystic fibrosis transmembrane conductance regulator.

## Competing interests

The authors declare that they have no competing interests.

## Authors' contributions

AS performed experiments in all figures except Fig. 1d. CM and IAB contributed Fig. 1d and assisted in drafting the manuscript. NAC assisted in the design and implementation of the exposure apparatus, contributed to experimental design, and contributed to data analysis. JLK designed all studies, interpreted the data, and wrote the manuscript.
